# A Role for Fetal Hemoglobin and Maternal Immune IgG in Infant Resistance to *Plasmodium falciparum* Malaria

**DOI:** 10.1371/journal.pone.0014798

**Published:** 2011-04-12

**Authors:** Chanaki Amaratunga, Tatiana M. Lopera-Mesa, Nathaniel J. Brittain, Rushina Cholera, Takayuki Arie, Hisashi Fujioka, Jeffrey R. Keefer, Rick M. Fairhurst

**Affiliations:** 1 Laboratory of Malaria and Vector Research, National Institute of Allergy and Infectious Diseases, National Institutes of Health, Bethesda, Maryland, United States of America; 2 Department of Physics and Electronics, School of Engineering, Osaka Prefecture University, Osaka, Japan; 3 Department of Pharmacology, Case Western Reserve University, Cleveland, Ohio, United States of America; 4 Division of Pediatric Hematology, Department of Pediatrics, Johns Hopkins School of Medicine, Baltimore, Maryland, United States of America; Agency for Science, Technology and Research (A*STAR), Singapore

## Abstract

**Background:**

In Africa, infant susceptibility to *Plasmodium falciparum* malaria increases substantially as fetal hemoglobin (HbF) and maternal immune IgG disappear from circulation. During the first few months of life, however, resistance to malaria is evidenced by extremely low parasitemias, the absence of fever, and the almost complete lack of severe disease. This resistance has previously been attributed in part to poor parasite growth in HbF-containing red blood cells (RBCs). A specific role for maternal immune IgG in infant resistance to malaria has been hypothesized but not yet identified.

**Methods and Findings:**

We found that *P. falciparum* parasites invade and develop normally in fetal (cord blood, CB) RBCs, which contain up to 95% HbF. However, these parasitized CB RBCs are impaired in their binding to human microvascular endothelial cells (MVECs), monocytes, and nonparasitized RBCs – cytoadherence interactions that have been implicated in the development of high parasite densities and the symptoms of malaria. Abnormal display of the parasite's cytoadherence antigen *P. falciparum* erythrocyte membrane protein-1 (PfEMP-1) on CB RBCs accounts for these findings and is reminiscent of that on HbC and HbS RBCs. IgG purified from the plasma of immune Malian adults almost completely abolishes the adherence of parasitized CB RBCs to MVECs.

**Conclusions:**

Our data suggest a model of malaria protection in which HbF and maternal IgG act cooperatively to impair the cytoadherence of parasitized RBCs in the first few months of life. In highly malarious areas of Africa, an infant's contemporaneous expression of HbC or HbS and development of an immune IgG repertoire may effectively reconstitute the waning protective effects of HbF and maternal immune IgG, thereby extending the malaria resistance of infancy into early childhood.

## Introduction

In Africa, resistance to *Plasmodium falciparum* malaria in the first few months of life is evidenced by extremely low parasitemias, the absence of fever, and the almost complete lack of severe disease [1]. Infant susceptibility to malaria then increases substantially as fetal hemoglobin (HbF) and maternal immune IgG disappear from circulation. Fetal red blood cells (RBCs), which contain HbF (α_2_γ_2_), have higher affinity for oxygen than adult RBCs, which contain hemoglobin A (HbA; α_2_β_2_), and this facilitates transfer of oxygen from the maternal to the fetal circulation. The switch from production of γ to β globin begins *in utero* and results in the linear decline of HbF in the fetal RBC population, such that HbF levels of 50–95% at birth decline to <5% by three months [2]. Levels of maternal IgG, which protect the mother from high-density parasitemia and malaria symptoms, are similar in maternal and cord blood at birth [3], and can be expected to also decline markedly in infants during this time period.

Infant resistance to malaria has previously been attributed to poor parasite growth in HbF-containing RBCs. While several studies have established that *P. falciparum* parasites readily invade cord blood (CB) RBCs [4], [5], [6], the presence of HbF in three RBC types (CB, infant, and adult hereditary persistence of fetal hemoglobin, HPFH) was believed to restrict parasite growth [4], [5], [6], [7]. Biochemical explanations for these findings were provided by studies that concluded that the ability of HbF-containing RBCs to handle the oxidative stress imposed by developing parasites is impaired [7], or that HbF is inefficiently digested by *P. falciparum* hemoglobinases [8]. While malaria resistance in infants has also been attributed to IgG acquired from immune mothers, support for this hypothesis is lacking. For example, most studies have failed to detect positive correlations between levels of parasite-specific maternal antibodies and measures of disease susceptibility in infants, including time to first parasite infection, density of parasites in the blood, and incidence of febrile episodes [9].

To improve our understanding of malaria pathogenesis and immunity, we sought to identify the mechanisms that confer such high levels of malaria resistance to infants. Our findings that *P. falciparum* parasites invade and develop normally in HbF-containing RBCs suggested an alternative mechanism of malaria resistance by HbF. We found that HbF impairs the binding of parasitized RBCs to human microvascular endothelial cells (MVECs), monocytes, and nonparasitized erythrocytes – cytoadherence interactions that contribute to the development of high parasite densities and the symptoms of malaria. Abnormal display of the parasite's cytoadherence antigen *P. falciparum* erythrocyte membrane protein-1 (PfEMP-1) on HbF RBCs correlates with these findings and is similar to that on HbC and HbS RBCs [10], [11]. We also found that IgG purified from the plasma of immune Malian adults markedly reduces the adherence of parasitized CB RBCs to MVECs. Taken together, these data suggest a model of malaria protection in which HbF and maternal PfEMP-1-specific IgG act cooperatively to impair the cytoadherence of parasitized RBCs in the first few months of life. HbS and HbC may thus have been naturally selected for their ability to reconstitute the waning protective effects of HbF in highly malarious areas of Africa, where IgG repertoires specific for PfEMP-1 variants are rapidly acquired.

## Methods

### Ethics Statement

Blood collections were approved by the Institutional Review Boards of the National Institute of Allergy and Infectious Diseases, the National Heart, Lung and Blood Institute, the Johns Hopkins School of Medicine, and the Ethics Committee of the Faculty of Medicine, Pharmacy, and Odontostomatology at the University of Bamako. All volunteers gave written informed consent.

### Erythrocytes

Adult HbA-containing (AA) and CB RBC samples were drawn into Vacutainers® containing acid-citrate-dextrose anticoagulant. CB samples were obtained from term deliveries and CB hemolysates contained 79%–100% HbF. After removing buffy coat leukocytes, RBCs were washed three times with RPMI-1640 (Invitrogen, Carlsbad, CA) and stored at 50% hematocrit at 4°C prior to use (within 4–36 h of blood draw). Hemoglobin types were determined by HPLC (D-10 Instrument, Bio-Rad, Hercules, CA). In all experiments, AA and CB (or HPFH) RBCs were simultaneously obtained and processed in parallel. The HPFH RBCs (containing 100% HbF) were obtained from a single adult donor.

### Parasite culture

*P. falciparum* lines (3D7, 7G8, FVO, GB4, MC/R+, TM284, FCR-3, A4tres) were cultured in O+ RBCs at 5% hematocrit in complete medium (CM; RPMI-1640 supplemented with 25 mg/mL HEPES, 2 mg/mL sodium bicarbonate, 50 µg/mL gentamicin, and either 10% heat-inactivated human AB+ serum or 0.5% Albumax II (Gibco-BRL, Grand Island, NY)). Knobby parasite lines were maintained by periodic gelatin flotation. Cultures were maintained at 37°C in a humidified atmosphere of 5% CO_2_ in air and media were changed every 8–24 h. Trophozoite-infected RBCs containing paramagnetic hemozoin were enriched to >95% purity by magnetic separation (Miltenyi Biotec, Auburn, CA), inoculated into AA and CB (or HPFH) RBC samples, and cultured at 1–2% hematocrit as above. In all experiments, parasitized RBCs were assayed after a single cycle of invasion to the ring stage (∼18 h) or development to the trophozoite stage (∼40 h). To avoid the confounding effects of *in vitro* RBC senescence on parasite invasion and development, we did not monitor parasite growth in subsequent cycles.

### Invasion and development assays

Mature parasitized RBCs were inoculated into 5 mL CM containing 5×10^8^ AA or CB erythrocytes. The numbers of ring parasitized RBCs per 1000 RBCs were counted at 18 h. Five *P. falciparum* lines (7G8, FVO, GB4, MC/R+, TM284) were tested in 4 AA and 6 CB RBC samples. At ∼40 h, mature parasitized RBCs were stained with ethidium bromide (2 µg/mL) at room temperature for 30 min and quantified by flow cytometry.

### Endothelial cell adherence assay

Adult dermal human microvascular endothelial cells (MVECs; Cambrex Biosciences, Walkersville, MD) were maintained in the manufacturer's EGM2-MV medium and grown on LabTek CC-2 coated 8-well chamber slides (Nalge Nunc International, Rochester, NY) to ∼50% confluency at 37°C in a humidified atmosphere of 5% CO_2_. Mature parasitized RBCs were adjusted to 5–20% parasitemia and 0.5–1% hematocrit by the addition of nonparasitized RBCs in binding media (BM; RPMI-1640, 0.5% BSA, pH 6.7). Adherent endothelial cells were washed with BM and then incubated with 150 µL of the parasitized RBC suspension for 1 h at ambient temperature with constant orbital agitation (100 rpm). In some experiments, these incubations were carried out in the presence of various concentrations of purified non-immune and immune IgG. After parasite suspensions were removed from each well, slides were washed by dipping 4 times in BM at 37°C, fixed in 2% glutaraldehyde at ambient temperature for 2 h, and stained in 10% Giemsa for 60 min. In each experiment, the number of parasitized RBCs bound to ∼700 endothelial cells was counted from duplicate wells. These counts were then expressed as the number of parasitized RBCs bound to 100 MVECs.

### Purification of IgG from human plasma

Serum was obtained from non-immune or immune individuals (i.e., Malian adults with ‘disease-controlling immunity’). IgG was purified from pooled serum samples by Protein G PLUS gel (Pierce, Rockford, IL) according to the manufacturer's directions. Eluted IgG was dialyzed in RPMI-1640, concentrated to approximately 80 mg/mL, and sterilized with a 0.22 µm filter (Millipore, Billerica, MA). Purified IgG was pre-adsorbed with human A positive and B positive RBCs (25 µL of packed erythrocytes per 1 mL of sample) and then stored at 4°C for immediate use or at −80°C. IgG was used in cytoadherence assays at final concentrations of 0.5–10 mg/mL.

### Monocyte adherence assay

CD14+ monocytes (Cambrex Biosciences) were plated onto CC2 Lab-Tek chamber slides (Nalge Nunc International) at 4×10^5^ cells per well and cultured for 48 h in RPMI 1640 containing 25 mM HEPES, 50 µg/mL gentamicin, and 10% fetal bovine serum at 37°C in an atmosphere of 5% CO_2_. AA and CB RBCs infected with *P. falciparum* trophozoites (3D7, A4tres, FCR-3) were purified by magnetic separation, adjusted to 10–15% parasitemia and 1% hematocrit as previously described. Adherent monocytes were washed with binding media and incubated with 150 µL of the parasitized RBC suspension for 1 h at ambient temperature with gentle orbital agitation. The parasite suspension was removed from each well and the slides were gently washed by dipping four times in binding media. Slides were dried and stained using Hema 3 (Fischer Scientific). Adherence was measured by counting the number of parasitized RBCs bound to a minimum of 700 monocytes from duplicate wells. These counts were then expressed as the number of parasitized RBCs bound to 100 monocytes.

### Rosetting

MC/R^+^ and TM284 parasites were cultured in AA and CB RBCs as above to 5% to 10% parasitemia. RBCs containing mature parasites were identified by their refractive hemozoin pigment. Rosettes were identified as parasitized RBCs to which three or more nonparasitized RBCs closely adhered. The rosette frequency was determined by dividing the total number of rosettes by >200 parasitized RBCs examined.

### Flow cytometry

Rat or rabbit polyclonal antisera raised against PfEMP-1 variants expressed by the *P. falciparum* lines FVO, MC/R+, and A4tres were kindly provided by Morris Makobongo and Dror Baruch (Malaria Vaccine Development Branch, NIAID). Samples of RBCs containing synchronized trophozoites (1.5×10^6^ cells; 1% parasitemia) were stained with various dilutions of antiserum in FACS staining buffer (FSB; PBS, 2% FBS, 0.1% sodium azide) for 45 min at room temperature and washed twice with FSB. Samples were then incubated with Alexa 488-conjugated anti-rat or anti-rabbit IgG (Molecular Probes, Inc., Eugene, OR) and ethidium bromide (2 µg/mL) at room temperature for 30 min and washed twice with FSB. A FACSort instrument (Becton-Dickinson, San Jose, CA) and FlowJo software (Tree Star, Inc., Ashland, OR) were used to acquire and analyze 250,000 to 500,000 events from each sample.

### Surface immunofluorescence/confocal microscopy

Parasitized RBCs (5 µl packed cell volume) were added to 50 µl rat polyclonal antiserum (1∶10 dilution, MC/R+; 1∶500 dilution, FVO) for 1 h at ambient temperature and washed. Bound antibody was detected with Alexa 488-conjugated anti-rat IgG (Molecular Probes, Inc.). Images were collected using a TCS-SP2 AOBS confocal microscope (Leica Microsystems, Wetzlar, Germany) using a 63X oil immersion objective NA 1.4, zoom 6. The confocal pinhole was set to 0.9 Airy units to ensure maximum resolution. Alexa 488 fluorescence was excited using an argon laser at 488 nm. 3-dimensional reconstructions were made using sequential sections through the sample with a z increment of 0.12 µm. Images were deconvoluted and processed using Leica TCS (version 2.1374), Imaris 4.1 (3-D reconstructions) (Bitplane AG), Huygens Essentials (deconvolution) (SVI) and Adobe Photoshop CS (Adobe Systems).

### Atomic force microscopy

Samples of parasitized RBCs for atomic force microscopy (AFM) imaging were prepared as described [11], [12]. A Bioscope AFM (Veeco Instruments, Santa Barbara, CA) on a wide-field Axiovert 200 fluorescence microscope (Carl Zeiss, Inc., Thornwood, NY) was optimized to image the surface topography of RBCs and to identify the parasite stage within an individual AFM-imaged RBC. The X and Y piezoelectric scanners of the Bioscope AFM were disconnected. A custom built closed-loop XY scanner stage (nPoint, Inc., Madison, WI) was used to minimize scanning artifacts and thermal drift of the scanner for improved image accuracy. AFM was performed in tapping mode in air using Nanosensors pointprobe tips (Nanosensors, Switzerland) with a cantilever resonance frequency of 327–397 kHz. Topographic and error signal (amplitude) images were collected simultaneously. Parasites were stained with YOYO-1 fluorescent nucleic acid staining reagent (Molecular Probes, Inc.). Bright field and fluorescent images were collected with a chilled CCD video camera (Model C5985, Hamamatsu Photonic Systems, Bridgewater, NJ). Image-Pro Plus version 5.0 (Media Cybernetics, Silver Spring, MD) was used to merge these images to allow the unambiguous identification of the parasite stage.

### Transmission electron microscopy

Parasitized RBCs were processed as described [13]. Briefly, samples were fixed with 2.5% glutaraldehyde in 0.05 M phosphate buffer, pH 7.4 containing 4% sucrose for 2 h and then postfixed in 1% osmium tetroxide for 1 h. After a 30-min *en bloc* stain with 1% aqueous uranyl acetate, the cells were dehydrated in ascending concentrations of ethanol and embedded in Epon 812. Ultrathin sections were stained with 2% uranyl acetate in 50% methanol and lead citrate and then examined in a Zeiss CEM902 electron microscope (Oberkochen, Germany).

### Statistical analysis

In assays of invasion, development, cytoadherence, and rosetting, results from parasitized AA and CB (or HPFH) RBCs were compared using the non-parametric Mann-Whitney test (GraphPad Software, San Diego, CA). PfEMP-1 levels on the surface of parasitized AA and CB erythrocytes were compared using the paired *t* test. In all comparisons, 2-tailed *P* values were calculated.

## Results

### *P. falciparum* parasites invade and develop normally in HbF-containing RBCs

**Figure 1:**
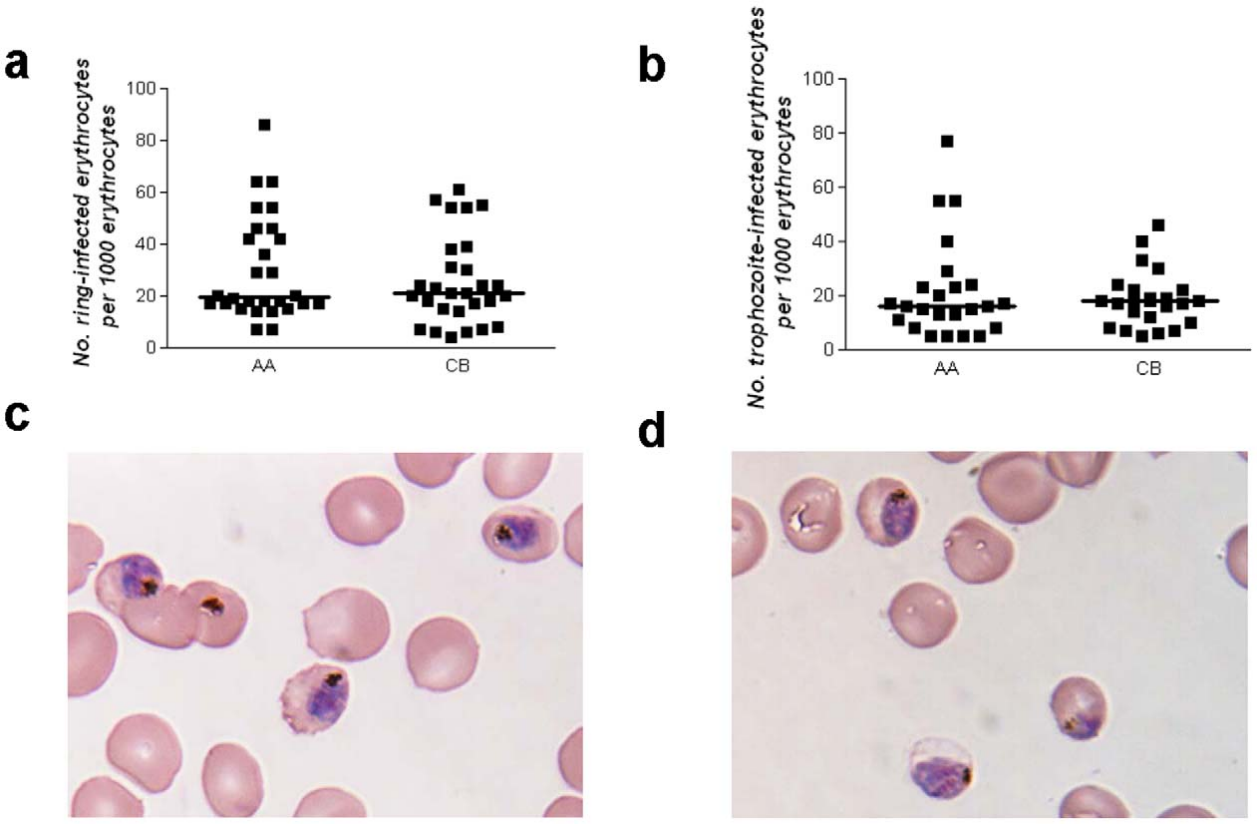
Invasion and development of *P. falciparum* in RBCs containing HbF. **a**, *P. falciparum* invasion of RBCs. The numbers of ring-infected RBCs per 1000 AA or CB RBCs are shown. Median values are indicated by horizontal bars. **b**, *P. falciparum* development in RBCs. The numbers of trophozoite-infected RBCs per 1000 AA or CB RBCs are shown. Median values are indicated by horizontal bars. Results in **a** and **b** were obtained from 5 parasite lines (7G8, GB4, MC/R+, FVO, TM284) and multiple blood donors (4AA, 6CB) (not all combinations tested). **c, d**, The photomicrographs show mature parasites in AA (**c**) and HPFH (**d**) RBCs (containing 100% HbF) that are morphologically normal and synchronously developed.

To investigate how HbF might impair parasite multiplication, we examined the invasion and development of five *P. falciparum* lines in AA and CB RBCs. Approximately 18 hours after infection, the number of ring-infected RBCs per 1000 RBCs (counted by light microscopy) did not differ significantly between AA and CB RBCs (median (range); 20 (7–86) for AA samples *vs.* 22 (4–61) for CB samples, *P* = 0.68, *N* = 30) (**Fig. 1a** **, Table S1**). Approximately 24 hours later, the number of trophozoite-infected RBCs per 1000 RBCs (quantified by flow cytometry) did not differ significantly between CB and AA RBCs (median (range); 16 (5–77) for AA samples *vs.* 18 (5–46) for CB samples; *P* = 0.69, *N* = 23) (**Fig. 1b** **, Table S1**). We observed neither morphological abnormalities nor growth restriction as ring forms matured into trophozoites and schizonts, even in RBCs containing 100% HbF from an adult with HPFH (**Figs. 1c, d**). Using transmission electron microscopy, we found that >99% of 480 trophozoite-infected CB RBCs appeared viable (i.e., there was neither nuclear condensation nor loss of membranous organelle integrity) (not shown). These data indicate that HbF-containing RBCs support robust invasion and development of *P. falciparum*.

### HbF impairs the cytoadherence of *P. falciparum*-infected RBCs

*P. falciparum* expresses PfEMP-1 cytoadherence proteins and concentrates them in knob-like protrusions on the RBC surface, where they mediate binding to a variety of host cells. The sequestration of parasitized RBCs in microvessels (i.e., ‘sequestration’) is associated with life-threatening manifestations of malaria, including the clinical syndrome of cerebral malaria [14], [15], [16], [17]. Sequestration is believed to contribute to high parasite densities by enabling large numbers of mature parasitized RBCs to avoid clearance from the blood stream by the spleen [18]. The importance of sequestration to parasite survival is suggested by animal studies in which the spleen modulates the *in vivo* sequestration and *in vitro* cytoadherence properties of *P. falciparum*-infected RBCs [19], [20], and by studies showing that mature parasitized RBCs are efficiently removed by *ex vivo*-perfused human spleens [21]. Sequestration not only contributes to the development of high parasite densities but also leads to microvascular inflammation and endothelial dysfunction [22], [23], [24].

**Figure 2:**
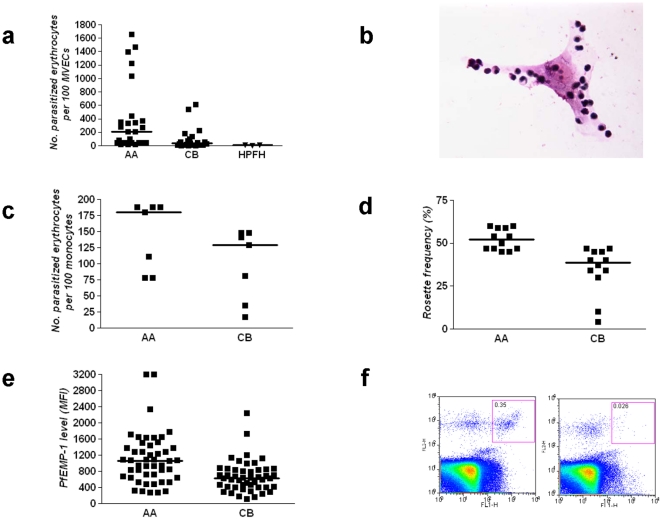
Cytoadherence and surface PfEMP-1 levels of parasitized RBCs containing HbF. **a**, Adherence of parasitized RBCs to MVECs. The numbers of parasitized AA, CB, and HPFH RBCs adhering to 100 MVECs are shown. Median values are indicated by horizontal bars. Results were obtained from 4 parasite lines (3D7, 7G8, A4tres, FCR-3), multiple blood donors (7AA, 10CB, 1HPFH), and 5 endothelial cell lots (not all combinations tested). **b**, The photomicrograph shows the adherence of parasitized AA RBCs to MVECs. **c**, Adherence of parasitized RBCs to blood monocytes. The numbers of parasitized AA and CB RBCs adhering to 100 monocytes are shown. Median values are indicated by horizontal bars. Results were obtained from 3 parasite lines (3D7, A4tres, FCR-3), multiple blood donors (4AA, 7CB), and 4 monocyte donors (not all combinations tested). **d**, Rosette frequencies of parasitized RBCs. Median values are indicated by horizontal bars. Results were obtained from 2 parasite lines (MC/R+, TM284) and multiple blood donors (4AA, 7CB) (not all combinations tested). **e**, PfEMP-1 expression levels (median fluorescence intensities, MFI) on the surface of parasitized RBCs. Mean values are indicated by horizontal bars. Results were obtained from 2 parasite lines (MC/R+, FVO), multiple blood donors (4AA, 7CB), and various concentrations of 2 antisera. **f**, Flow cytometry analysis of PfEMP-1 expression on AA (left panel) and HPFH (right panel) RBCs infected with the FVO *P. falciparum* line. Parasitized RBCs reactive to PfEMP-1-specific antiserum appear as a clustered population in the upper right quadrant (left panel).

Because sequestration is linked to parasite multiplication and microvascular inflammation, we tested *P. falciparum*-infected RBCs for their adherence to MVECs, which express the major host adherence receptor CD36. Relative to parasitized AA RBCs, parasitized CB RBCs showed an 82% reduction in adherence to MVECs (median (range) number of parasitized RBCs per 100 MVECs; 205 (21–1657) for AA samples vs. 37 (0.1–614) for CB samples, *P* = 0.0013, *N* = 24) (**Figs. 2a, b** **, Table S1**). Parasitized HPFH RBCs (containing 100% HbF) showed a 88% reduction in adherence to MVECs (median (range); 100 (19–1228) for AA samples vs. 12 (0.4–13) for HPFH samples, *P* = 0.10, *N* = 3) (**Fig. 2a**). These data suggest that HbF may reduce parasite multiplication rates and dampen microvascular inflammation in vivo by weakening the adherence of parasitized RBCs to MVECs.

The interaction between PfEMP-1 on parasitized RBCs and CD36 on monocytes leads to monocyte activation and monokine production *in vitro* [25], [26], [27]. Monocyte activation during *P. falciparum* infection is believed to produce elevated levels of TNF and other monokines implicated in the pathogenesis of malaria [28]. To explore whether HbF might impair monocyte activation, we tested the effect of HbF on the adherence of parasitized RBCs to monocytes. We found that parasitized CB RBCs showed a 28% reduction in adherence to monocytes relative to parasitized AA RBCs (median (range) number of parasitized RBCs per 100 monocytes; 180 (78–188) for AA samples vs. 129 (17–148) for CB samples, *P* = 0.200, *N* = 7) (**Fig. 2c** **, Table S1**). The level of reduced adhesion of parasitized CB RBCs to monocytes is less than that to MVECs. This is probably due to the higher levels of CD36 on monocytes compared to MVECs (unpublished data). These data suggest that HbF may weaken the interaction between parasite RBCs and blood monocytes *in vivo*.

Rosette formation, the attachment of nonparasitized RBCs to parasitized RBCs, is also PfEMP-1-mediated and is associated with malaria severity in Africa [29], [30]. Rosettes are proposed to impede microvascular blood flow and to contribute to local hypoxia-induced inflammation. In Saimiri monkeys, rosettes have also been associated with elevated parasite multiplication rates *in vivo* [31]. In this setting, rosette formation may enable short-lived merozoites to efficiently recognize and invade suitable host RBCs upon schizont rupture. To test how HbF affects this pathogenetic interaction, we infected RBCs with rosetting *P. falciparum* lines and cultured them for ∼40 hours to allow for maturation to the trophozoite stage, which expresses PfEMP-1. We then determined the proportion of 200 parasitized RBCs that bound three or more nonparasitized RBCs (i.e., rosette frequency) and found that rosette frequencies were 25% lower in parasitized CB RBCs than in parasitized AA RBCs (median (range) rosette frequency; 52% (45%–60%) for AA samples *vs.* 39% (4%–47%) for CB samples, *P* = 0.0003, *N* = 12) (**Fig. 2d** **, Table S1**). These data suggest that HbF impairs rosetting, which may reduce parasite multiplication rate and the degree of microvascular obstruction *in vivo*.

### Abnormal PfEMP-1 and knob display on the surface of *P. falciparum*-infected RBCs

**Figure 3:**
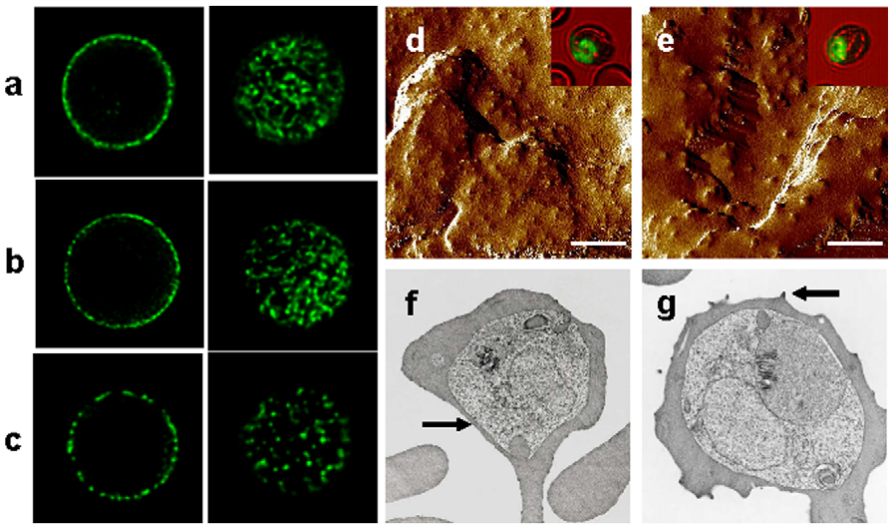
Distributions of PfEMP-1 signals and knobs on the surface of parasitized AA and CB RBCs. **a–c**, Confocal cross-sections through the mid-plane (left column) or maximum intensity projections of *z* stacks through the upper cell surface (right column) of trophozoite-infected RBCs probed with a polyclonal antiserum against PfEMP-1 (FVO line). **a**, PfEMP-1 fluorescence patterns typical of a parasitized AA RBC. **b**, Fluorescence patterns from a parasitized CB RBC, similar to those of parasitized AA RBCs. **c**, Irregular, patchy PfEMP-1 distribution on a parasitized CB RBC. **d,e**, Atomic force micrographs of CB RBCs infected with the FVO *P. falciparum* line, showing normal AA-like (**d**) or abnormal (**e**) knob appearances. The mean (± SEM) width of 15 randomly selected knobs in panels **d** and **e** were 50.3 nm (±3.4 nm) and 96.4 nm (±6.7 nm), respectively. Scale bar  = 1 µm. Transmission electron micrographs of CB RBCs infected with the FVO *P. falciparum* line also showed populations with normal (**f**) or abnormal (**g**) knob appearances. Comparison atomic force and transmission electron micrographs of parasitized HbC and HbS RBCs can be found in references 9, 10, and 11. Arrows indicate examples of normal (**f**) and abnormal (**g**) knobs.

Reduced expression of PfEMP-1 on the surface of parasitized RBCs containing the malaria-protective HbC or HbS mutations is associated with impaired cytoadherence [10], [11]. Using specific antisera in a flow cytometry assay, we found that parasitized CB RBCs had a 40% reduction in surface PfEMP-1 levels relative to parasitized AA RBCs (mean ± SEM; 1111±93 for AA samples *vs.* 674±56 for CB samples, *P*<0.0001, *N* = 48) (**Fig. 2e** **, Table S1**). We were unable to detect PfEMP-1 on the surface of parasitized HPFH RBCs containing 100% HbF (**Fig. 2f**). We also used an immunofluorescence assay (IFA) to detect PfEMP-1 on the surface of parasitized CB RBCs. By confocal microscopy, we found that parasitized AA RBCs had a relatively homogeneous distribution of PfEMP-1 (**Fig. 3a**). Signals from parasitized CB RBCs, however, were heterogeneous, with some cells showing AA-like patterns (**Fig. 3b**) and others showing dim and uneven patterns (**Fig. 3c**) reminiscent of HbC and HbS RBCs [10], [11].

PfEMP-1 molecules are concentrated on knob-like protrusions that mediate contact between parasitized RBCs and MVECs or monocytes [14], [27]. Since PfEMP-1 proteins are anchored in knobs, alterations in knob assembly can affect the amount and distribution of PfEMP-1 [10], [11], [32]. To determine whether HbF is associated with aberrant knob morphology and distribution, we examined the surface of parasitized CB RBCs. Atomic force micrographs showed populations of parasitized CB RBCs expressing fine, regularly distributed knobs (**Fig. 3d**) characteristic of parasitized AA RBCs, and populations expressing large, widely separated knobs (**Fig. 3e**) reminiscent of HbC and HbS RBCs [11], [12]. The concordant appearance of these knob distributions with the patterns of IFA signals (**Figs. 3b, c**) is consistent with widely dispersed anchoring and irregular concentrations of PfEMP-1 at the surface of parasitized CB RBCs. Transmission electron micrographs of parasitized CB RBCs confirmed the presence of normal (**Fig. 3f**) and abnormal (**Fig. 3g**) knob morphologies. Knobs were found to be abnormal or entirely absent on the surface of parasitized HPFH RBCs (**Figure S1**).

### Immune IgG impairs the cytoadherence of *P. falciparum*-infected RBCs

**Figure 4:**
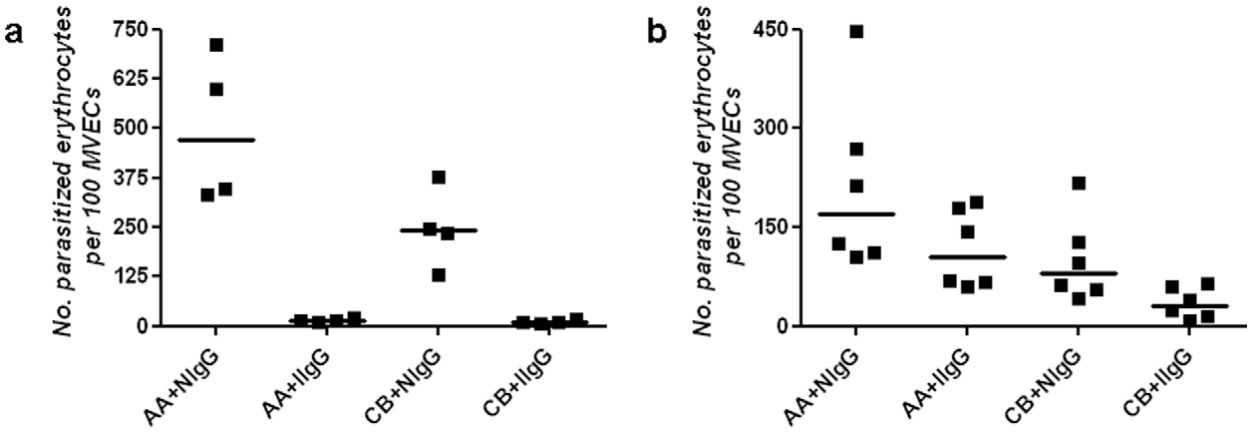
Adherence of parasitized RBCs to MVECs in the presence of immune IgG. The numbers of parasitized AA and CB RBCs adhering to 100 MVECs in the presence of either non-immune or immune IgG are shown. Comparisons were made in parallel using IgG at final concentrations of either 10 mg/mL (**a**) or 0.5–6 mg/mL (**b**). Median values are indicated by horizontal bars. Results were obtained from 2 parasite lines (3D7, FCR-3), multiple blood donors (6AA, 6CB), and 4 endothelial cell lots. For each combination of parasite line and RBC sample tested, the numbers of parasitized RBCs bound to 100 MVECs are shown in Table S2.

‘Disease-controlling immunity,’ which eventually develops in adults residing permanently in malaria-endemic areas of Africa, enables individuals to tolerate *P. falciparum* parasitemias without developing the symptoms of malaria. Since disease-controlling immunity to malaria is associated with the development of PfEMP-1-specific antibodies [33], we hypothesized that PfEMP-1-specific IgG works cooperatively with HbF to impair cytoadherence *in vivo*. To explore this possibility, we purified IgG from pooled plasma obtained from adult residents of a malaria-endemic area of Mali. The preparations of immune IgG agglutinated parasitized RBCs (**not shown**), indicating that they recognized PfEMP-1 (and perhaps other proteins) expressed on the surface of these cells. Relative to non-immune IgG, immune IgG (at physiologically-relevant levels of 10 mg/mL) essentially abrogated the binding of parasitized AA and CB RBCs to MVECs (median (range) number of parasitized RBCs per 100 MVECs; 472.5 (334–712) *vs.* 13 (9–23) for AA samples, *P* = 0.029, *N* = 4; 242 (129–378) *vs.* 9.5 (6–19) for CB samples, *P* = 0.029, *N* = 4) (**Fig. 4a** **, Table S2**). To mimic the waning concentrations of maternal IgG in the infant circulation, we also performed cytoadherence assays in the presence of 0.5–6 mg/mL immune IgG. Relative to non-immune IgG, immune IgG (at 0.5–6 mg/mL) reduced the binding of parasitized AA and CB RBCs to MVECs (median (range) number of parasitized RBCs per 100 MVECs; 169.5 (105–448) *vs.* 106 (59–189) for AA samples, *P* = 0.18, *N* = 6; 79.5 (43–218) *vs.* 31.5 (8–65) for CB samples, *P* = 0.04, *N* = 6) (**Fig. 4a** **, Table S2**). These data indicate that lower concentrations (<10 mg/mL) of immune IgG are able to effect greater reductions in the cytoadherence of parasitized CB RBCs than parasitized AA RBCs, suggesting that PfEMP-1-specific IgG works cooperatively with HbF to reduce cytoadherence *in vivo*.

## Discussion

Infant resistance to malaria has been associated temporally with the presence of HbF-containing RBCs and transplacentally-acquired, maternal immune IgG in the circulation. To date, the mechanisms by which these two host factors might produce such dramatic reductions in parasite density and malaria incidence have not been established. In considering candidate mechanisms of malaria resistance by HbF and immune IgG, we reasoned that they should be consistent with two well-documented *in vivo* observations. First, infant RBCs are inherently able to support the invasion and development of *P. falciparum*, as evidenced by patent parasitemias (up to 10,000/µL) in the first few months of life [9]. Second, passive transfer studies conducted 50 years ago showed that immune IgG – but not nonimmune IgG – could rapidly drive down parasitemias in African children with malaria [34]. While invasion-inhibitory antibodies may contribute to this phenomenon, the large and rapid decreases in parasitemias suggest a particularly important role for PfEMP-1-specific IgGs. By binding PfEMP-1, such IgGs may prevent ring-parasitized RBCs from sequestering and promote their efficient removal from the circulation by the spleen. Accordingly, we hypothesized that HbF and PfEMP-1-specific IgG work cooperatively in infants to impair the cytoadherence of parasitized RBCs and prevent sequestration, thus limiting the ability of *P. falciparum* to multiply and cause inflammation.

In testing this hypothesis, we found that *P. falciparum* parasites invade and develop normally in CB and HPFH RBCs, which contain up to 100% HbF. This suggested that the presence of HbF does not restrict parasite multiplication *in vivo* by killing intraerythrocytic parasites. To explain how infants maintain extremely low parasitemias (often detectable only by PCR methods) and remain asymptomatic with relatively higher parasitemias (up to 10,000/µL), we investigated several cytoadherence interactions involved in the development of high parasite densities and the symptoms of malaria. We found that parasitized CB RBCs are impaired in their binding to MVECs, monocytes, and nonparasitized RBCs. Abnormal display of PfEMP-1 and knobs on the surface of CB RBCs correlated with these findings and is reminiscent of that on HbC and HbS RBCs [10], [11]. IgG (at physiological levels of 10 mg/mL) purified from the plasma of immune Malian adults almost completely abolished the adherence of parasitized AA and CB RBCs to MVECs. At lower doses (up to 6 mg/mL), immune IgG effected greater reductions in the cytoadherence of parasitized CB RBCs than parasitized AA RBCs, suggesting that HbF and maternal immune IgG work cooperatively to impair cytoadherence *in vivo*.

We propose that HbF and PfEMP-1-specific IgG substantially attenuate parasite virulence by weakening cytoadherence *in vivo*, which prevents the sequestration of most parasitized RBCs at the late ring stage (only early ring forms are observed circulating in the bloodstream) and thus enables their removal from the bloodstream by the spleen. Lower avidity interactions with host cells might also mitigate the inflammatory potential of the remaining sequestered parasites. By these mechanisms, HbF and maternal immune IgG could produce the dramatic reductions in parasite densities and malaria episodes observed in young infants – consistent with the proposed cooperative effects of HbS and acquired immunity [11], [35]. This model suggests that HbF and other hemoglobin variants might confer less protection against malaria in low-transmission settings where humoral immunity is not transplacentally or naturally acquired. This hypothesis is further strengthened by reports of non-immune HbS children with high parasitemias and severe malaria [36], and could be more rigorously tested by studying non-immune hemoglobinopathic individuals who acquire malaria. Our findings indicate that measurements of PfEMP-1-specific antibody levels are likely to be particularly relevant and informative in studies of infant immunity to malaria [37], [38], [39].

By altering PfEMP-1 display, HbF impairs cytoadherence by a mechanism similar to that of HbC and HbS [10], [11], two hemoglobin variants for which malaria protection is well documented [40], [41], [42], [43], [44], [45]. Like HbC and HbS RBCs [46], CB RBCs contain elevated levels of membrane-associated hemichromes [47] and IgG (**not shown**). These and other shared characteristics might interfere with the trafficking of PfEMP-1 to the CB RBC membrane. In addition, the waning protective effects of maternal immune IgG and HbF might be reconstituted by the contemporaneous development of humoral immunity and expression of HbC and HbS in some African infants, which would cooperate to extend malaria resistance into their early childhood. Our data thus provide a rationale for testing whether hydroxyurea and other HbF-inducing agents can be used as prophylaxis against severe and fatal malaria in very young ‘wildtype’ children who lack protective polymorphisms. Our findings also raise the possibility that polymorphisms that produce unstable hemoglobin (HbC, HbS, HbE, alpha- and beta-thalassemias, G6PD deficiency) confer malaria resistance by a common mechanism. Cooperative interactions between cytoadherence-inhibiting IgG (e.g., PfEMP-1-specific IgG) and diverse hemoglobin variants suggest that therapeutics and vaccines that disrupt PfEMP-1-mediated cytoadherence phenomena will reduce the morbidity and mortality of malaria.

## Supporting Information

Table S1 Relative red blood cell (RBC) invasion and development, cytoadherence and rosetting, and PfEMP-1 expression on parasitized AA and CB RBCs: data separated out by P. falciparum line.
(0.02 MB DOCX)

Table S2 Relative adherence of parasitized AA and CB red blood cells (RBCs) to microvascular endothelial cells (MVECs) in the presence of nonimmune IgG (NIgG) or immune IgG (IIgG).
(0.01 MB DOCX)

Figure S1 Morphology and distribution of knobs on the surface of parasitized HPFH RBCs.
(5.98 MB TIF)
