# Supplementary material for: A Role for Fetal Hemoglobin and Maternal Immune IgG in Infant Resistance to Plasmodium falciparum Malaria
Source: PLoS One. 2011 Apr 12;6(4):e14798. doi: 10.1371/journal.pone.0014798 (PMC3075246; doi:10.1371/journal.pone.0014798)
Supplement: Table S1 — Relative red blood cell (RBC) invasion and development, cytoadherence and rosetting, and PfEMP-1 expression on parasitized AA and CB RBCs: data separated out by P. falciparum line. (0.02 MB DOCX) [file pone.0014798.s001.docx]

| **Assay** | ***P. falciparum* line** | | | | | | | | |
| --- | --- | --- | --- | --- | --- | --- | --- | --- | --- |
|  | **All *P.f.*** | **MC/R+** | **FVO** | **TM284** | **7G8** | **GB4** | **3D7** | **A4tres** | **FCR3** |
|  | | | | | | | | | |
| **Invasion^1^** |  |  |  |  |  |  |  |  |  |
| **CB** | 22 (4-61) | 18 (6-30) | 38 (18-61) | 8 (4-24) | 34 (7-54) | 20 (17-23) |  |  |  |
| **AA** | 20 (7-86) | 20 (14-36) | 42 (17-86) | 17 (7-19) | 34 (15-54) | 18 (18-29) |  |  |  |
| ***N*** | 30 | 7 | 11 | 5 | 4 | 3 |  |  |  |
| ***P* value*** | 0.68 | 0.56 | 0.36 | 1.00 | 0.66 | 1.00 |  |  |  |
|  | | | | | | | | | |
| **Development^2^** |  |  |  |  |  |  |  |  |  |
| **CB** | 18 (5-46) | 10 (5-19) | 30 (17-46) | 13 (6-22) | 18 (17-18) | 18 (16-22) |  |  |  |
| **AA** | 16 (5-77) | 13 (5-24) | 23 (17-77) | 10 (5-15) | 16 (16-16) | 8 (8-29) |  |  |  |
| ***N*** | 23 | 7 | 7 | 4 | 2 | 3 |  |  |  |
| ***P* value** | 0.69 | 0.61 | 0.70 | 0.31 | NC | 0.66 |  |  |  |
|  | | | | | | | | | |
| **EC adherence^3^** |  |  |  |  |  |  |  |  |  |
| **CB** | 37 (0.1-614) |  |  |  | 7.5 (7-8) |  | 41 (2-614) | 112 (0.1-223) | 37 (13-46) |
| **AA** | 205 (21-1657) |  |  |  | 47 (47-47) |  | 205 (21-1657) | 724 (47-1400) | 1040 (268-1228) |
| ***N*** | 24 |  |  |  | 2 |  | 17 | 2 | 3 |
| ***P* value** | 0.001 |  |  |  | NC |  | 0.02 | NC | 0.10 |
|  | | | | | | | | | |
| **MO adherence^4^** |  |  |  |  |  |  |  |  |  |
| **CB** | 129 (17-148) |  |  |  |  |  | 144 (81-148) |  |  |
| **AA** | 180 (78-188) |  |  |  |  |  | 188 (111-188) |  |  |
| ***N*** | 7 |  |  |  |  |  | 4 |  |  |
| ***P* value** | 0.20 |  |  |  |  |  | 0.19 |  |  |
|  | | | | | | | | | |
| **Rosetting^5^** |  |  |  |  |  |  |  |  |  |
| **CB** | 39 (4-47) | 40 (30-47) |  | 34 (4-45) |  |  |  |  |  |
| **AA** | 52 (45-60) | 59 (50-60) |  | 47 (45-47) |  |  |  |  |  |
| ***N*** | 12 | 7 |  | 5 |  |  |  |  |  |
| ***P* value** | 0.0003 | 0.002 |  | 0.02 |  |  |  |  |  |
|  | | | | | | | | | |
| **PfEMP-1 levels^6^** |  |  |  |  |  |  |  |  |  |
| **CB** | 674 (56) | 925 (115) | 536 (45) |  |  |  |  |  |  |
| **AA** | 1111 (93) | 1479 (176) | 909 (89) |  |  |  |  |  |  |
| ***N*** | 48 | 17 | 31 |  |  |  |  |  |  |
| ***P* value**** | <0.0001 | 0.006 | 0.0008 |  |  |  |  |  |  |
|  | | | | | | | | | |

* Two-tailed *P* values calculated by Mann-Whitney test; NC = not calculated, due to fewer than 3 values in each group.

** Two-tailed *P* values calculated by paired *t* test.

^1^ Invasion expressed as the median (range) of the number of ring-infected RBCs per 1000 RBCs.

^2^ Intraerythrocytic development expressed as the median (range) of the number of parasitized RBCs per 1000 RBCs.

^3^ Cytoadherence expressed as the median (range) number of parasitized RBCs bound to 100 endothelial cells.

^4^ Cytoadherence expressed as the median (range) number of parasitized RBCs bound to 100 monocytes.

^5^ Rosetting expressed as the median (range) rosette frequency (%).

^6^ PfEMP-1 levels expressed as mean (SEM) median fluorescence intensity.
