# Supplementary material for: A Role for Fetal Hemoglobin and Maternal Immune IgG in Infant Resistance to Plasmodium falciparum Malaria
Source: PLoS One. 2011 Apr 12;6(4):e14798. doi: 10.1371/journal.pone.0014798 (PMC3075246; doi:10.1371/journal.pone.0014798)
Supplement: Table S2 — Relative adherence of parasitized AA and CB red blood cells (RBCs) to microvascular endothelial cells (MVECs) in the presence of nonimmune IgG (NIgG) or immune IgG (IIgG). (0.01 MB DOCX) [file pone.0014798.s002.docx]

|  |  |  | **10 ug/ml IgG** | | | |
| --- | --- | --- | --- | --- | --- | --- |
| ***P. falciparum*** | **AA RBC** | **CB RBC** | **AA + NIgG** | **AA + IIgG** | **CB + NIgG** | **CB + IIgG** |
| FCR-3 | AA-2 | CB-11 | 712 | 23 | 378 | 19 |
| FCR-3 | AA-1 | CB-10 | 597 | 14 | 248 | 9 |
| 3D7 | AA-4 | CB-13 | 348 | 9 | 236 | 10 |
| FCR-3 | AA-3 | CB-12 | 334 | 12 | 129 | 6 |
|  |  |  | **<10ug/mL IgG** | | | |
| ***P. falciparum*** | **AA RBC** | **CB RBC** | **AA + NIgG** | **AA + IIgG** | **CB + NIgG** | **CB + IIgG** |
| 3D7 | AA-6 | CB-15 | 448 | 189 | 218 | 61 |
| 3D7 | AA-5 | CB-14 | 269 | 180 | 127 | 65 |
| FCR-3 | AA-5 | CB-14 | 214 | 143 | 96 | 40 |
| FCR-3 | AA-6 | CB-15 | 125 | 67 | 43 | 8 |
| 3D7 | AA-5 | CB-14 | 112 | 69 | 63 | 23 |
| FCR-3 | AA-5 | CB-14 | 105 | 59 | 56 | 14 |
|  |  |  |  |  |  |  |
