# Supplementary figures and images for: A Role for Fetal Hemoglobin and Maternal Immune IgG in Infant Resistance to Plasmodium falciparum Malaria
Source: PLoS One. 2011 Apr 12;6(4):e14798. doi: 10.1371/journal.pone.0014798 (PMC3075246; doi:10.1371/journal.pone.0014798)

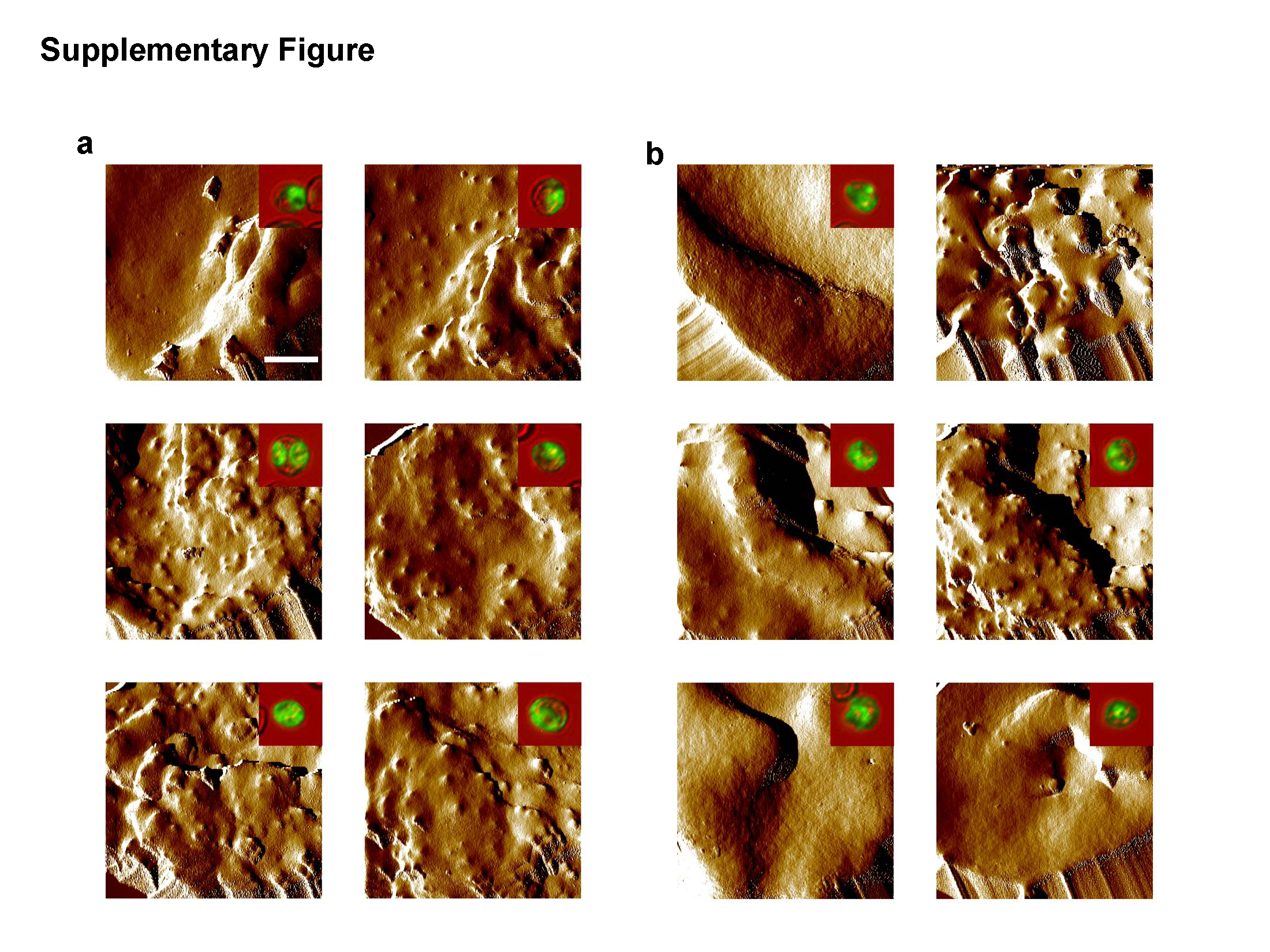

Supplement: Figure S1 — Morphology and distribution of knobs on the surface of parasitized HPFH RBCs. (5.98 MB TIF) [file pone.0014798.s003.tif]
